# Linear atrophoderma of Moulin

**DOI:** 10.1111/1346-8138.17268

**Published:** 2024-05-06

**Authors:** Andrea Paradisi, Irina Ciobotariu, Laura Quattrini, Francesco Ricci, Ketty Peris

**Affiliations:** ^1^ Dermatologia, Dipartimento di Medicina e Chirurgia Traslazionale Università Cattolica del Sacro Cuore Roma Italy; ^2^ UOC di Dermatologia, Dipartimento di Scienze Mediche e Chirurgiche Fondazione Policlinico Universitario A. Gemelli – IRCCS Roma Italy; ^3^ Dermatology Department IDI‐IRCCS Roma Italy

Linear atrophoderma of Moulin (LAM) is a rare, acquired dermatosis that follows Blaschko's lines.^1^ Although its exact pathogenesis is unclear, its distribution is believed to reflect genetic mosaicism.^2^

Linear atrophoderma of Moulin onset is typically sudden, most frequently during adolescence or childhood. It is not commonly preceded by inflammation or followed by sclerosis, and is characterized by hyperpigmented, slightly atrophic patches on the trunk or extremities. The lesions show a unilateral distribution, although bilateral involvement has been reported.^3^, ^4^ In the first months, LAM usually progresses as linear atrophic lesions, then progression ceases and the lesions persist.

We present the case of a 16‐year‐old male with a 3‐year history of progressive, asymptomatic, depressed linear lesions on the left upper trunk that became more defined over a short period.

The disease arose on the posterior trunk as pigmented atrophic plaques extending laterally from the paravertebral region toward the left shoulder, at the level of T3 of the thoracic spine. After 2–3 months the plaques became well defined, but were asymptomatic with no sign of inflammation or induration of the skin (Figure 1a–d).

**FIGURE 1:**
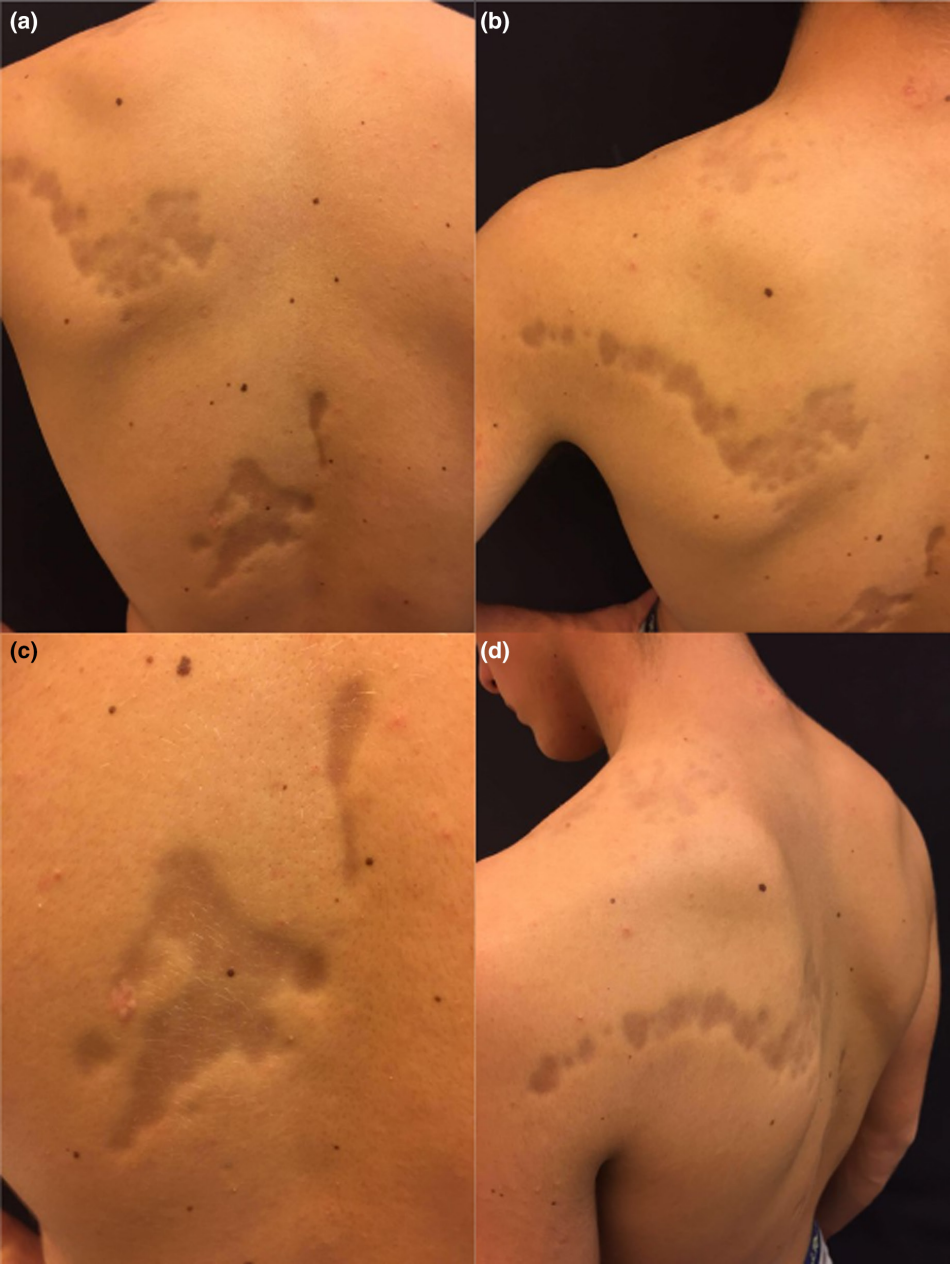
(a–d) Hyperpigmented, atrophic plaques on the upper trunk involving the left scapula and the paravertebral area.

There was no underlying disease. None in the patient's family had similar lesions.

On histopathology, the lesion showed epidermal loss of the rete ridges, mild hyperplasia of basal melanocytes, and a reduced dermal thickness with sparse lymphocytic infiltrate, vasodilatation, and slight thickening of the superficial collagen fibers (Figure S1).

There is no effective treatment for LAM, but a partial response to topical calcipotriol has been reported.^5^ It is debated whether LAM belongs to a spectrum including atrophoderma of Pasini and Pierini and linear scleroderma. However, despite some similarities, the different age at onset, distribution, histology, origin, development, and prognosis of LAM suggest that it is a separate disease and highlights the importance of recognizing its distinct clinical features.

## CONFLICT OF INTEREST STATEMENT

None declared.

## PATIENT CONSENT

Written patient consent was obtained for use of the images.

## Supporting information


Figure S1.
