# Supplementary material for: Linear atrophoderma of Moulin
Source: J Dermatol. 2024 May 6;51(6):e183–4. doi: 10.1111/1346-8138.17268 (PMC11483920; doi:10.1111/1346-8138.17268)
Supplement: Supplementary file 1 — Figure S1. [file JDE-51--s001.docx]

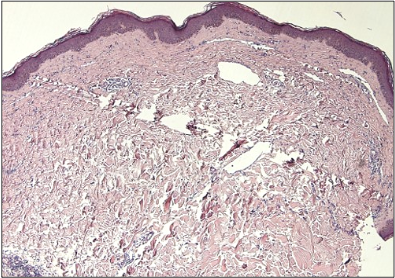


Figure S1. Histology revealing a slight reduction of the thickness of the dermis and subcutaneous tissue with more compact collagen and sparse inflammation (hematoxylin-eosin stain; original magnification: x100).
